# Core–Shell Microspheres with Encapsulated Gold Nanoparticle Carriers for Controlled Release of Anti-Cancer Drugs

**DOI:** 10.3390/jfb15100277

**Published:** 2024-09-24

**Authors:** Lin Guo, Qilong Zhao, Min Wang

**Affiliations:** 1 Department of Mechanical Engineering, The University of Hong Kong, Pokfulam Road, Hong Kong, China; guolin913@outlook.com; 2 Institute of Biomedical & Health Engineering, Shenzhen Institute of Advanced Technology (SIAT), Chinese Academy of Sciences (CAS), Shenzhen 518055, China

**Keywords:** drug delivery, core–shell microsphere, gold nanoparticle, coaxial electrospray, chemotherapy

## Abstract

Cancer is one of the major threats to human health and lives. However, effective cancer treatments remain a great challenge in clinical medicine. As a common approach for cancer treatment, chemotherapy has saved the life of millions of people; however, patients who have gone through chemotherapy often suffer from severe side effects owing to the inherent cytotoxicity of anti-cancer drugs. Stabilizing the blood concentration of an anti-cancer drug will reduce the occurrence or severity of side effects, and relies on using an appropriate drug delivery system (DDS) for achieving sustained or even on-demand drug delivery. However, this is still an unmet clinical challenge since the mainstay of anti-cancer drugs is small molecules, which tend to be diffused rapidly in the body, and conventional DDSs exhibit the burst release phenomenon. Here, we establish a class of DDSs based on biodegradable core–shell microspheres with encapsulated doxorubicin hydrochloride-loaded gold nanoparticles (DOX@Au@MSs), with the core–shell microspheres being made of poly(lactic-co-glycolic acid) in the current study. By harnessing the physical barrier of the biodegradable shell of core–shell microspheres, DOX@Au@MSs can provide a sustained release of the anti-cancer drug in the test duration (which is 21 days in the current study). Thanks to the photothermal properties of the encapsulated gold nanoparticle carriers, the core–shell biodegradable microspheres can be ruptured through remotely controlled near-infrared (NIR) light, thereby achieving an NIR-controlled triggered release of the anti-cancer drug. Furthermore, the route of the DOX-Au@MS-enabled controlled release of the anti-cancer drug can provide durable cancer cell ablation for the long period of 72 h.

## 1. Introduction

Each year, the number of global deaths from all types of cancers is estimated to be more than 9.7 million [1]. As a major threat to human health and lives, cancer has been catching extensive attention worldwide and such attention has led to the development of effective therapeutic approaches [2,3,4]. To date, chemotherapy remains one of the most common approaches for treating various types of cancers [5]. Despite numerous human lives being saved, chemotherapy still faces serious problems of high side effects as the mainstays of anti-cancer drugs have a high cytotoxicity, which can cause severe damages to normal organs or stimulate the immune system [6,7,8]. To alleviate the side effects of chemotherapy resulting from the dosage-dependent toxicity of anti-cancer drugs and yet maintain the therapeutic effectiveness, stabilizing appropriate blood concentrations of anti-cancer drugs is required; however, this cannot be achieved by direct administration of anti-cancer drugs due to their short half-life [9]. Therefore, it is urgent to develop new drug delivery systems (DDSs) capable of controlling the release of the anti-cancer drugs in a sustained manner or even on demand for avoiding the instantly ultra-high blood stream drug concentration.

Over the past decades, we have witnessed remarkable advances in developing DDSs for anti-cancer drugs [10,11], where nanocarrier-based DDSs are promising owing to their high surface-to-volume ratio that is beneficial for drug loading and passive targeting capability owing to the enhanced permeability and retention (EPR) effect [12,13,14,15,16,17,18]. Among the nanocarriers, gold nanoparticles (AuNPs) have shown obvious superiority thanks to their unique physicochemical properties, especially surface-enhanced Raman spectroscopic (SERS) activities and photothermal effects, which provide attractive combined therapeutic and diagnostic functions [16,17,18,19,20]. In addition, AuNPs are also capable of the high-efficiency loading of anti-cancer drugs regardless of the hydrophobic or hydrophilic nature of the drug, as well as of actively targeting cancer cells or tumor microenvironments, owing to their versatile surface functionalization [21,22,23], for promoting therapeutic efficiency. Nevertheless, anti-cancer drugs are mainly small molecules. Influenced by the rapid diffusion of drug molecules in the body and the small sizes of nanocarriers, anti-cancer drugs often display a burst release when delivering them using nanocarrier-based DDSs including AuNPs [24,25], impairing the efficiency and biosafety of chemotherapy.

**Figure 1:**
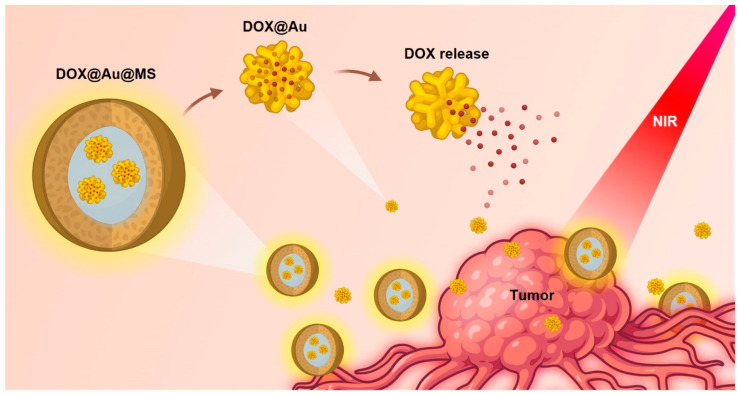
Schematic illustration showing the design and formation of DOX-Au@MSs for the controlled release of anti-cancer drugs and durable killing of cancer cells. Upon exposure to an NIR light, the DOX@Au@MSs would be ruptured, caused by photothermal effects of the AuNPs, subsequently releasing the DOX@Au and then the DOX in a spatiotemporally controllable manner.

In the current study, we develop a class of DDSs that encapsulate anti-cancer drug-loaded AuNPs within biodegradable core–shell microspheres (namely, DOX@Au@MSs), realizing improved sustained and near-infrared (NIR)-controlled release of a model anti-cancer drug, doxorubicin hydrochloride (DOX), by harnessing the physical barrier of the shell of core–shell structured microspheres and the photothermal properties of AuNPs (Figure 1). The DOX@Au@MSs are facilely fabricated via coaxial electrospraying, whose core–shell structures can be disrupted under an NIR irradiation to release the DOX-loaded AuNPs (DOX@Au), having implications of providing combined diagnostic and therapeutic functions for cancer treatment. Furthermore, the route of the DOX-Au@MS-enabled controlled release of the anti-cancer drug can provide durable cancer cell ablation for the long period of 72 h, holding promise of alleviating the side effects of chemotherapy.

## 2. Experimental Section

### 2.1. Materials

The anti-cancer drug, DOX in the hydrochloric salt form, was purchased from Mesochem, China. Tripolyphosphate (TPP) was supplied by Uni-Chem, UK. Folic acid (FA), dicyclohexylcarbodiimide (DCC), hydroxysuccinimide (NHS), chitosan (CS) with a low molecular weight (50,000–190,000 Da), rhodamine 6G (R6G) and chloroauric acid (HAuCl_4_·3H_2_O) were purchased from Sigma-Aldrich, St. Louis, MO, USA. Dimethyl sulfoxide (DMSO) and dichloromethane (DCM) were supplied by Fisher Scientific, USA. Poly(lactic-co-glycolic acid) (PLGA) with an LA:GA molecular ratio and an inherent viscosity of 0.6–0.8 dL/g of 50:50 (PLGA50/50) was supplied by Lakeshore Biomaterials, Birmingham, AL, USA. All the chemicals were used as received without further purification. We used deionized (DI) water in the experiment, which was obtained from a DI water producer (Model D12681, Barnstead International, Dubuque, IA, USA).

### 2.2. Fabrication of DOX@Au

We first synthesized the R6G-embedded AuNPs with capped FA and CS, with reported theranostic functions, including SERS activities and actively targeting capabilities, according to the established one-pot synthesis method previously developed by our group [26]. Briefly, a mixture solution of FA, DCC and NHS in DMSO was stirred at room temperature for 12 h in the dark, which was then slowly added with a CS solution and further stirred at room temperature for 16 h in the dark, resulting in FA-conjugated CS. An amount of 1.554 × 10^−5^ mol FA molecules could be conjugated, as reported by us previously [26]. We then mixed a 10 mL FA-conjugated CS solution with a 2 mL HAuCl_4_ aqueous solution (1.0 mM), which was then heated to 90 °C and stirred for 3 h for the reduction of AuCl_4_^−^. Afterwards, R6G was added, where the mixture solution was stirred at room temperature in the dark for 12 h, finally obtaining the theranostic AuNPs. We then dispersed the theranostic AuNPs within DOX solutions according to the formulations listed in Table 1, followed by the stirring at room temperature for 12 h. Subsequently, we added 0.1 mL solution of TPP (2 mg/mL, as a crosslinking agent) into the above mixture of liquids to assist drug absorption by ionic gelation. The AuNPs with different loading contents of DOX (Table 1) were obtained after centrifuging at 7000× *g* for 20 min and washing with DI water. The supernatants were collected to determine the loading efficiency by measuring the non-absorbed DOX via a spectrophotometer (Lambda 20, Perkin Elmer, Waltham, MA, USA) according to the established method [27].

### 2.3. Fabrication of DOX-Loaded Core–Shell Microspheres

Through coaxial electrospraying, we prepared two types of core–shell-structured microspheres for DOX delivery: (1) microspheres with encapsulated DOX@Au (namely, DOX@Au@MSs), and (2) microspheres with co-encapsulations of the theranostic AuNPs (i.e., the R6G-embedded AuNPs with capped FA and CS) and DOX (namely, DOX-Au@MSs). The coaxial electrospraying was performed according to the established methods introduced by our previous studies [28]. Briefly, we first prepared the inner liquids by adding different amounts of DOX into the AuNPs suspensions and the outer liquids by dissolving 5 *w*/*v*% PLGA50/50 in DCM, which were then fed via a coaxial nozzle at feeding rates of 0.763 and 2.03 mL/h, respectively. Electrospraying was performed at the high voltage of 15 kV, resulting in DOX-Au@MSs with different loading amounts of DOX, as listed in Table 2.

Using the DOX@Au with the optimal DOX loading efficiency, we also performed the coaxial electrospraying using the same processing parameters to fabricate the DOX@Au@MSs. All these microspherical samples were freeze-dried for 2 days to completely remove the solvents prior to following uses or investigations.

### 2.4. Morphological and Structural Characterizations

The morphologies and structures of the theranostic AuNPs, DOX-Au@MSs and DOX@Au@MSs were characterized by a transmission electron microscope (TEM, Tecnai G2 20S-TWIN, FEI, Portland, OR, USA), where the samples for the TEM observation were collected on carbon-film-coated copper grids and freeze-dried for 48 h. Additionally, we also characterized the morphologies of the DOX-Au@MSs and DOX@Au@MSs using a field-emission scanning electron microscope (SEM, LEO 1530, Gemini, Germany), where the samples for the SEM observation were coated with a thin layer of gold via sputtering.

### 2.5. In Vitro DOX Release Examination

To examine the release profile of DOX, we first immersed the DOX-loaded samples in the phosphate buffer saline (PBS, pH 7.4) at 37 ºC and then collected the supernatant at the pre-set time points, followed by the centrifuge at 13,000 rpm for 10 min. The samples of interest were irradiated by a NIR laser (785 nm wavelength) at a power density of 2 W cm^−2^ for 5 min. The concentration of DOX was measured by the UV–Vis spectrophotometer and calculated according to the absorption intensity at the wavelength of 470 nm and the calibration curve. The cumulative release curve of DOX for each sample was then plotted (n = 3 for experiments).

### 2.6. Cell Culture and Cell Viability Assay

We used HeLa cells as the model in our experiment, which was supplied by ATCC, USA. The Hela cells were cultured using Dulbecco’s modified eagle medium (DMEM) supplemented with 10.0 *v/v*% fetal bovine serum (FBS) and 100 units/mL penicillin–streptomycin (P/S) at T-75 cell culture flasks, maintained in a 37 °C incubator pumped with 5.0 *v/v*% CO_2_ atmosphere. In the cell viability assessment, we seeded the Hela cells in a 24-well tissue culture plate at the cell density of 3.0 × 10^4^ cells/well. Twenty-four hours later, we added the sterilized DOX@Au@MSs samples (~0.2 g) into each well and then stained the co-incubated cells at the pre-set time points using a Live/Dead viability kit (Molecular Probes, USA) according to the established methods. The stained cells were then observed under a fluorescence microscope (Fluoview FV 1000, Olympus, Tokyo, Japan). HeLa cells incubated with the DOX-free samples were used as a control. The viability of HeLa cells in the DOX@Au@MS-treated and control groups were compared according to representative fluorescent images with the assistance of the software Image J (n = 3 experiments).

### 2.7. Statistics

In this study, all numerical data were expressed as means ± standard deviation.

## 3. Results and Discussion

### 3.1. Formation of DOX@Au@MSs

**Figure 2:**
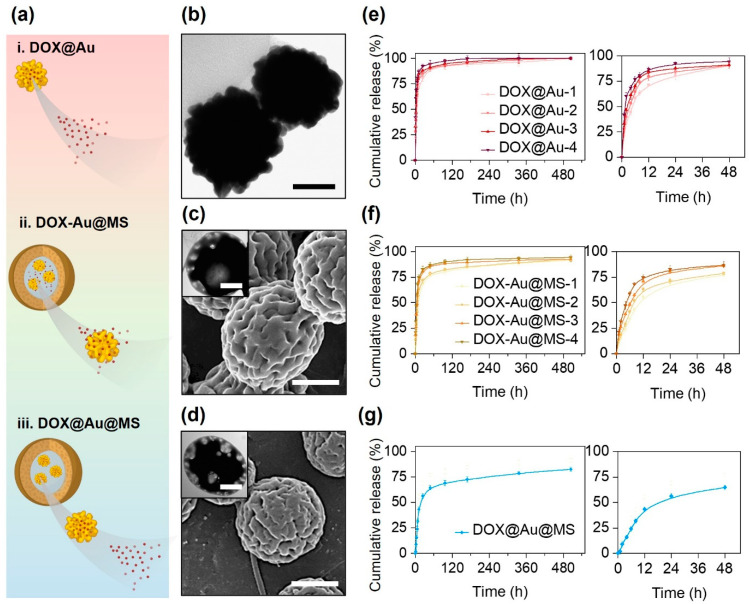
Different release behaviors of DOX from different types of delivery vehicles. (**a**) Schematically illustrating the differential release behaviors of DOX from diverse delivery vehicles. (**b**) A representative TEM image of DOX@Au. Scale bar: 50 nm. (**c**) A representative SEM image of DOX-Au@MSs, with an inset showing a representative TEM image of DOX-Au@MSs. Scale bar: 100 nm and 2 μm (inset). (**d**) A representative SEM image of DOX@Au@MSs, with an inset showing a representative TEM image of DOX@Au@MSs. Scale bar: 100 nm and 2 μm (inset). (**e**–**g**) Cumulative release behaviors of DOX from DOX@Au (**e**), DOX-Au@MSs (**f**) or DOX@Au@MSs (**g**), with the right curves showing the release within the initial 48 h.

In this study, we prepared three types of DOX DDS, i.e., DOX@Au, DOX-Au@MSs and DOX@Au@MSs (Figure 2a), aiming to compare the release of DOX from either nanocarriers (DOX@Au) or microcarriers (DOX-Au@MSs and DOX@Au@MSs).

As shown in Figure 2b, the DOX@Au possesses rough surface morphologies and highly branched nano-spherical structures, with an average diameter of around 80 nm, similar to the routine AuNPs without DOX loading reported in our previous studies [29]. This result suggests that the loading of DOX would not significantly affect the morphologies and structures of the original AuNPs, which are important for attaining high SERS activities [30]. We then measured the loading efficiency of DOX at varying DOX@Au nanocarriers, finding that the DOX@Au with a moderate amount of initially added DOX (0.4 mg) possessed the highest loading efficiency of 64.3 ± 5.1% (Table 3). These results are supposed to be arisen by the unsaturated adsorption at the low DOX concentration while competing adsorption at the high DOX concentrations. We next chose such DOX@Au with the optimal DOX loading in preparing the DOX@Au@MSs.

Through coaxial electrospraying, we then prepared the DOX-Au@MS and DOX@Au@MS microcarriers, whose morphologies and structures were characterized by SEM and TEM, as indicated in Figure 2c and Figure 2d, respectively. They showed similar microspherical structures of diameters both ranging from 2 to 5 μm, as well as the same core–shell structures that would offer the spaces for the encapsulation of AuNPs and DOX molecules [31].

### 3.2. DOX Controlled Release

As for the varying nanocarriers and microcarriers, we then compared the release behaviors of DOX from different DDSs via in vitro release examinations within a 21-day period of release time. According to the cumulative release curve (Figure 2e), we validated that the DOX release from the DOX@Au nanocarriers typically showed a burst release profile. With the increase in DOX loading, the faster release rates were presented, of which release kinetics followed a diffusion-controlled model [32]. For the DOX@Au with the optimal loading efficiency of DOX (with initially 0.4 mg DOX added), half of the dosage was released within the initial 4 h (Figure 2e, right). Over a period of 48 h, over 80% of the loaded DOX would be released. Such a burst release profile should be attributed to the high surface-to-volume ratio of the nanocarriers [15]; however, it might lead to risky side effects due to the instant excessive administrations of the toxic DOX molecules.

From the DOX-Au@MS microcarriers, the release kinetics of the DOX would be affected by not only molecular diffusion but also biodegradation of the PLGA50/50 microspherical shell. PLGA50/50 core–shell structured microspheres would undergo rapid swelling (in several hours) and gradual degradation (for several days to weeks) along with the hydrolysis of polyester chains in the hydrated environments, which would result in rupturing of the microspheres and thereby offer the routes for the release out of the entrapped molecules [31]. Accordingly, the microcarriers with co-encapsulation of AuNPs and DOX showed a slightly delayed release profile as compared to the DOX@Au nanocarriers (Figure 2f), as the PLGA50/50 microspherical shell would act as the physical barriers for delaying the molecular diffusion [33,34], where half of the dosage would be released within the initial 8 h and less than 80% of the loaded DOX would be released over 48 h (Figure 2f, right). When we further firstly loaded the DOX onto the AuNPs to be the DOX@Au at the optimal loading efficiency of DOX (with 0.4 mg DOX added) and then encapsulated the DOX@Au inside the coaxial electrosprayed PLGA50/50 microspheres, we found that the DOX release would be more sustained from the DOX@Au@MS microcarriers (Figure 2g) as the diffusion of the nanosized DOX@Au would be more difficult than that of the small molecular DOX. The release of the DOX from the DOX@Au@MSs exhibited the typical two-stage characteristics of a burst release within the initial 24 h and a nearly sustained release in the following 20 days, which were determined by the synergistic effects of PLGA50/50 microsphere degradation and DOX molecular diffusion. Notably, we found the period of time for the release of half the dosage increased to 12–24 h (Figure 2g, right). Such sustained and long-lasting anti-cancer drug release would be beneficial for eliminating the side effects of chemotherapy [11].

**Figure 3:**
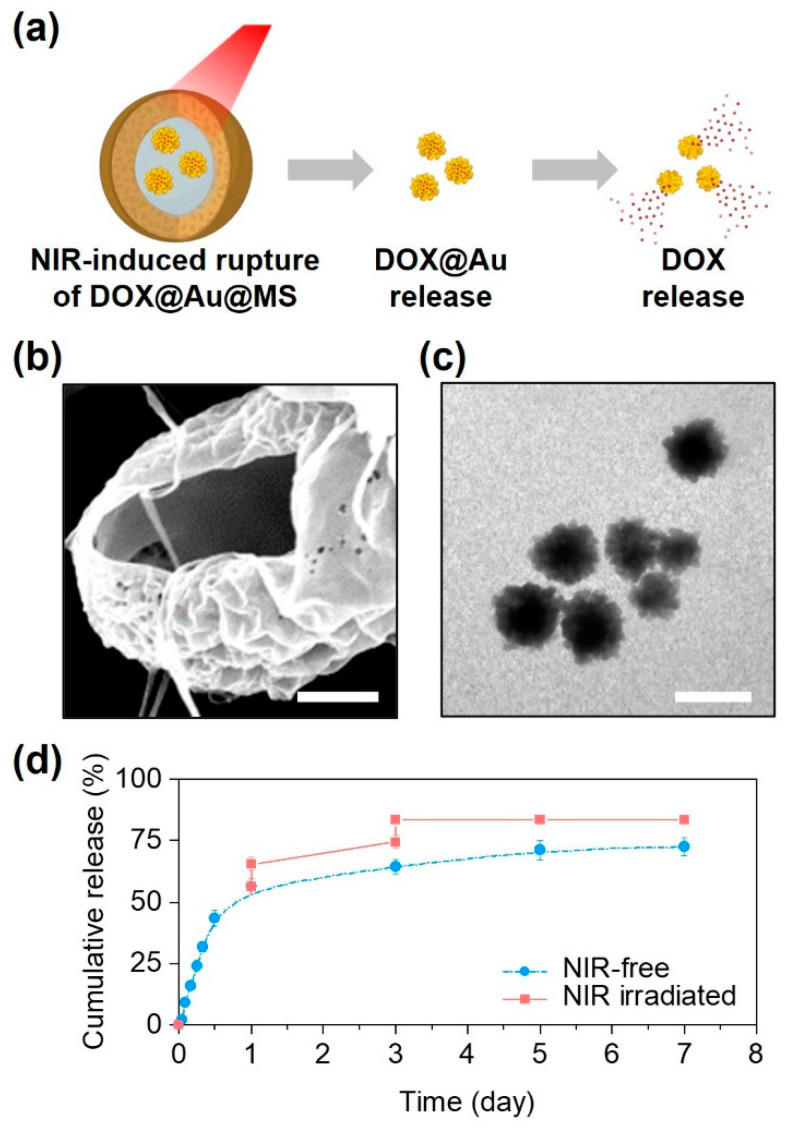
NIR-controlled microsphere rupture and DOX release. (**a**) Schematically illustrating the NIR-controlled release of DOX from the DOX@Au@MSs. (**b**) A representative SEM image showing the ruptured structure of the DOX@Au@MSs after NIR irradiation. Scale bar: 5 μm. (**c**) A representative TEM image showing the released DOX@Au from the DOX@Au@MSs after NIR irradiation. Scale bar: 100 nm. (**d**) Cumulative release behaviors of DOX from the DOX@Au@MSs with or without NIR irradiation.

Beyond sustained release, another notable outcome of the DOX@Au@MS microcarriers was a triggerable release profile in response to NIR irradiation (Figure 3a). Due to the reported photothermal effects of the AuNPs and relatively low melting temperature of the PLGA50/50 (about 90 ℃) [28,35], the intense heat generated by the embedded AuNPs under the NIR irradiation would lead to rupturing of the core–shell structures and the formation of large holes (Figure 3b). Interestingly, we could subsequently observe the controllable release of AuNPs from the microcarriers upon exposure to the NIR irradiation, while their morphologies and structures preserved well (Figure 3c), offering the possibilities of SERS-based cancer diagnostics and combinational photothermal therapy. In addition, we also found that the release profile could further be modulated by the NIR irradiation. As compared to the non-irradiated control, the DOX@Au@MSs under the NIR irradiation exerted at day 1 and day 3 showed a NIR-triggered release profile of DOX, by increasing nearly 70% of the DOX release contents (Figure 3d). We also noted that the effects of NIR irradiation on modulating the release profile of DOX tended to be limited at day 5 and day 7 as the amounts of encapsulated DOX@Au nanoparticles and the remaining DOX loading both decreased.

### 3.3. Durable Killing of Cancer Cells

**Figure 4:**
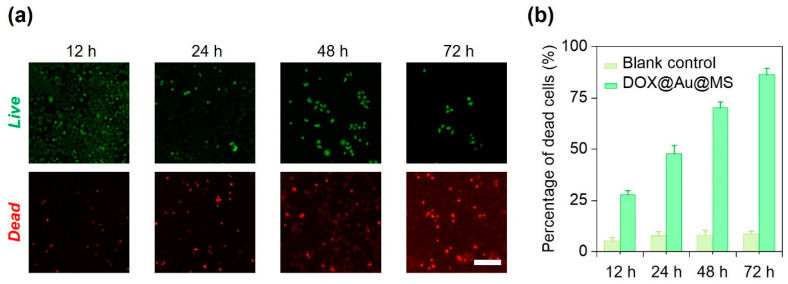
In vitro anti-cancer performance of DOX@Au@MSs. (**a**) Representative fluorescence images showing the HeLa cells treated with the DOX@Au@MSs for different amounts of time. Scale bar: 5 μm. (**b**) Statistics of the percentages of dead cells for the HeLa cells treated with the DOX@Au@MSs or the DOX-free control over different amounts of time.

We next used HeLa cells as the model to examine the effectiveness of the DOX@Au@MS microcarriers in killing the cancer cells via controlled release of the anti-cancer drugs. We cultured HeLa cells in a 24-well tissue culture plate and then co-incubated them with the sterilized DOX@Au@MS microcarriers or the DOX-free microspheres (as the blank control) for up to 72 h. At 12, 24, 48 and 72 h, we stained the HeLa cells with the Live/Dead cell staining kit and evaluated the cell viability of each group. As shown in Figure 4a, the DOX@Au@MS leads to gradual death of the HeLa cells within a period of 72 h, as indicated by increasing number of dead cells. We then quantitively evaluated the percentage of dead cells between the DOX@Au@MSs and the blank control groups, validating definitive effects of the controlled release anti-cancer drugs on the durable killing of cancer cells, while the blank control would not lead to death of the HeLa cells (Figure 3b). With 12, 24, 48 and 72 h co-incubation, the Hela cells were killed by 27.7%, 47.8%, 70.1% and 86.3%, respectively. The DOX@Au@MSs showed long-term anti-cancer efficiency, which should be attributed by sustained release of anti-cancer drugs. Considering that clinical uses of DOX, usually by ways of direct use or formulating into PEGylated liposomal DOX, encounter problems of a short half-life (directly used DOX: 1–3 h; PEGylated liposomal DOX: ~20 h) [36], the DOX@Au@MSs with a long half-life (over 24 h) and the potential of long-term anti-cancer efficiency (at least 74 h) are promising for addressing the problems. However, the pharmacokinetics, therapeutic effectiveness and biosafety of DOX@Au@MSs should be further investigated in rodent or even non-human primate models in the future.

## 4. Conclusions

In summary, we develop a new class of DDSs in the form of core–shell structured microspheres with encapsulated DOX-loaded AuNPs for controlled release of the anti-cancer drug. As compared to DOX-loaded AuNP nanocarriers (half-dosage release time: 4 h) and the DOX/AuNP-co-encapsulated microcarriers (half-dosage release time: 8 h), the DOX@Au@MS microcarriers showed much-improved sustained release profiles of the anti-cancer drug within a duration of 21 days, as well as an obviously prolonged half-dosage release time of 12–24 h. Notably, the DOX@Au@MS microcarriers further exhibited triggered release characteristics of DOX in response to an NIR light, where the heat generated by the encapsulated AuNPs under NIR irradiation could rupture the shell of core–shell-structured microcarriers to subsequently release the DOX@Au. Thanks to the controlled release behaviors of the anti-cancer drug, the DOX@Au@MS microcarriers realized durable effects in killing cancer cells for at least 72 h. Such DDSs that enable sustained and triggered drug release hold promise for not only alleviating the side effects of chemotherapy but also for other biomedical applications such as tissue engineering and regenerative medicine [37,38,39].

## Figures and Tables

**Table 1:** Formulations for preparing the DOX@Au.

Designations	AuNPs (mmol)	DOX (mg)
DOX@Au-1	1.0	0.2
DOX@Au-2	1.0	0.4
DOX@Au-3	1.0	0.6
DOX@Au-4	1.0	0.8

**Table 2:** Formulations for preparing the DOX-Au@MSs.

Designations	AuNPs (mmol)	DOX (mg)
DOX-Au@MS-1	1.0	0.2
DOX-Au@MS-2	1.0	0.4
DOX-Au@MS-3	1.0	0.6
DOX-Au@MS-4	1.0	0.8

**Table 3:** Loading efficiency of DOX on the DOX@Au.

Designations	Loading Efficiency (%)
DOX@Au-1	59.0 ± 4.2
DOX@Au-2	64.3 ± 5.1
DOX@Au-3	55.0 ± 4.1
DOX@Au-4	49.8 ± 3.7

## Data Availability

The original contributions presented in the study are included in the article, further inquiries can be directed to the corresponding authors.
